# Differentiation of *Bacillus thuringiensis* From *Bacillus* *cereus* Group Using a Unique Marker Based on Real-Time PCR

**DOI:** 10.3389/fmicb.2019.00883

**Published:** 2019-04-30

**Authors:** Shuai Wei, Ramachandran Chelliah, Byung-Jae Park, Se-Hun Kim, Fereidoun Forghani, Min Seok Cho, Dong-Suk Park, Yong-Guo Jin, Deog-Hwan Oh

**Affiliations:** ^1^Department of Medical Biomaterials Engineering, Institute of Bioscience and Biotechnology, Kangwon National University, Chuncheon, South Korea; ^2^Department of Food Science and Biotechnology, Kangwon National University, Chuncheon, South Korea; ^3^Center for Food Safety, College of Agricultural and Environmental Sciences, University of Georgia, Griffin, GA, United States; ^4^Department of Agricultural Biotechnology, National Academy of Agricultural Science, Rural Development Administration, Jeonju, South Korea; ^5^National Research and Development Center for Egg Processing, College of Food Science and Technology, Huazhong Agricultural University, Wuhan, China

**Keywords:** *B. thuringiensis*, transcriptional regulator, crystal protein, kimbap, spinach, lettuce

## Abstract

The efficiency of a novel biomarker (the transcriptional regulator, *XRE*) was tested and evaluated in differentiating *Bacillus thuringiensis* from *Bacillus cereus* group species in environmental and spiked samples based on PCR and real-time PCR. Totally 120 strains, representing two bacterial groups, *B. cereus* group and non-*Bacillus* sp., were used to evaluate the performance of *XRE* and crystal protein (*cry*2, an existing biomarker). Further, three diverse samples (kimbap, lettuce, and spinach) were inoculated with *B. thuringiensis* and prominent biomarkers *XRE* and *cry*2 were used as targets. Direct analysis of the detection results for the pure cultures of *B. cereus* group wild-types, references and type strains revealed an accuracy rate of 97.5% targeting *XRE*, and 83.3% targeting *cry*2. The real-time PCR was constructed with a *R*^2^-value of 0.993. For the artificially contaminated samples, a concentration of 10^3^ CFU/g of *B. thuringiensis* in spiked food samples could be detected using real-time PCR targeting *XRE.* A good performance was obtained with *XRE* in discriminating *B. thuringiensis* from *B. cereus* groups, as well as detecting *B. thuringiensis* in spiked food samples with PCR or real-time PCR. Therefore, this real-time PCR targeting X*RE* can be used as a dependable and promising tool to identify *B. thuringiensis* in foods.

## Introduction

*Bacillus thuringiensis* and the other 7 species of spore forming Gram positive bacteria, including *B. cereus, B. cytotoxicus, B. anthracis, B. pseudomycoides, B. weihenstephanensis, B. toyonensis*, and *B. mycoides*are, are primarily detected in soil. Because these bacteria are highly similar in genotype and phenotype, the bacteria are classified as *B. cereus* group in taxonomy (Park et al., 2007). *B. cereus* and *B. thuringiensis* are highly detectable in foods since they are observed in raw materials from agricultural soil during cultivation and distribution. In addition, these two bacteria are usually not discriminated in clinical diagnostics. *B. cereus* is a second risk priority group of foodborne illness in fresh agricultural products, and its contamination is one of the major problems in vegetables (Felício et al., 2015). In particular, *B. thuringiensis* was used as a pesticide in the cultivation of certain crops and it is well-known as microbial insecticides that have been used to reduce the amount of chemical pesticides (Azmi et al., 2015).

*B. thuringiensis* produces insecticidal proteins, which are the main type of Crystalline (*Cry*) proteins (Kutasi et al., 2016). Conversely, actively growing vegetative cells that lack crystal production lead to non-toxic effects. The δ-endotoxins mainly contains Cytolytic (*Cyt)* and *Cry* (Elleuch et al., 2015; Rao et al., 2015). However, *Cyt* and *Cry* possess different sequence homologies even though they consist of similar modes of action toward cell lysis, which lead to permanent damage of the insect midgut (Adang et al., 2014).

The structural variation of four δ-endotoxins (*Cry*1, *Cry*2, *Cry*3, and *Cyt*2Ah) was observed by X-ray crystallography (Dehury et al., 2013). These genes are identified in transgenic cotton and other vegetables, which are considerably effective in controlling pests. Designing a biomarker for the translated product varies with the different categories of *cry* protein; hence, the detection will be complex. The 16S rRNA gene sequences based on universal primers showed high similarity (>99%) index between *B. cereus* and *B. thuringiensis* (Böhm et al., 2015), which cannot be classified using genetic and phenotypic assays (Peng et al., 2015). Further, there has been a discussion since 2000 regarding whether the entire *B. cereus* group should be treated as a complex species of diverse bacteria (Helgason et al., 2000; Bartoszewicz and Marjańska, 2017). There are also suggestions that phylogeny of these bacteria better fits to their ecological properties (psychrotolerance, virulence) than to taxonomic affiliation (Drewnowska and Swiecicka, 2013; Bartoszewicz and Marjańska, 2017). To resolve this problem, we targeted *XRE* to detect *B. thuringiensis*, which controls the major type of crystal protein production.

These two bacteria are highly similar in biochemical results (Böhm et al., 2015), and genetic properties (Osman et al., 2015). Cho et al. (2015) also reported that the genetic and phenotypic properties between these bacteria are barely distinguishable. Furthermore, Pfrunder et al. reported that *B. cereus* group species cannot be reliably identified using classical biotyping (Pfrunder et al., 2016). Based on these, the biochemical experiments might not be enough for differentiation. Instead, the presence of an insecticidal crystal protein was used as a distinguished characteristic to differentiate these bacteria (Ekino et al., 2014; Wei et al., 2018).

Some of the factors that differentiate these two bacteria are based on their pathogenicity in samples. *B. cereus* causes gastrointestinal disorder, while *B. thuringiensis* has also been involved in epidemics of diarrhea. As reported, the distinguishing characteristic of *B. thuringiensis* is the presence of insecticidal crystal proteins (δ-endotoxin) encrypted by *cry* genes (Osman et al., 2015; Albright et al., 2016). Since the identification method using the biomarker for differentiating *B. cereus* group is complex and time consuming, a highly efficient biomarker is urgently needed to replace the previous ones that gave a lower efficiency and sometimes even showed false results. Based on the two specific genes, the transcriptional regulator and crystal protein genes for *B. thuringiensis* (Porcar and Juárez-Pérez, 2003; Han et al., 2006), the current study was performed to inspect and compare the efficiency of the designed biomarker (*XRE*) to that of the existing crystal protein marker (*cry*2) in identifying *B. thuringiensis* from *B. cereus* group strains (Bcg) and non-*B. cereus* group strains (non-B) in foods. *cry*2is the most common crystal protein present in *B. thuringiensis* (Liang et al., 2011; Palma et al., 2014).

## Materials and Methods

### Bacteria Cultures Preparation

All the 120 strains, including 111 of Bcg and 9 non-B, were obtained from bacterial collections in the United States and South Korea (Supplementary Table 1). Among the collections, *B. thuringiensis* ATCC 10792 (type strain) was used for the optimization of experimental conditions of conventional PCR and real-time PCR. All the strains were thawed at the room temperature (25°C) and streaked on the nutrient agar (Difco, MI, United States). After culturing for 24 h at 35°C in the incubator, a pure colony was picked and inoculated in tryptic soy broth (Difco, MI, United States) and overnight cultures were used for subsequent experiments.

### Primer Design and DNA Isolation

The respective primers targeting *XRE* for differentiating *B. thuringiensis* (Table 1) were designed according to the following sequence in Bacillus thuringiensis ATCC 10792 (Gene – 3664610–3665047) using Primer Express^^®^^ Software (Version 3.0.1) from Applied Biosystems located in CA, United States and synthesized by Bioneer Corporation from Daejeon, Korea. The performance of designed *XRE* was compared with *cry*2 (Ben-Dov et al., 1997). After 24 h enrichment in TSB, an aliquot of 1 mL of bacteria was subjected to the genomic DNA extraction using PrepMan^^®^^ Ultra Sample Preparation Reagent (Applied Biosystems, CA, United States). DNA concentrations were measured by Eppendorf BioSpectrometer^^®^^ fluorescence (Eppendorf, Hamburg, Germany) and adjusted to 10 ng/μL. DNA samples were stored at -20°C before use.

**Table 1:** Oligonucleotide primer sequences used in this study.

Target gene	Primer sequence (5′ → 3′)	Product Size (bp)	References
Transcriptional regulator (*XRE*)	AAG ATA TTG CAA GCG GTA AGA T	246	This study
	GTT TTG TTT CAG CAT TCC AGT AA		
Crystal Protein (*cry*2)	GTT ATT CTT AAT GCA GAT GAA TGG G	700	Ben-Dov et al., 1997
	CGG ATA AAA TAA TCT GGG AAA TAG T		


### Conventional PCR and Real-Time PCR Assay

The conventional PCR were prepared with a C1000 Touch^TM^ Thermal Cycler from Bio-Rad located in Hemel Hempstead, United Kingdom. The reaction tube for the PCR used Accu-Power^^®^^ Pyro Hot Start Taq PCR Pre-Mix from Bioneer Corporation located in Daejeon, Korea and a volume of 20 μL, including 1 μL of each primer (10 pmoL/μL) and 2 μL of DNA. Amplification conditions of the conventional PCR for transcriptional regulator (*XRE*) were: initial denaturation at 95°C for 5 min, followed by 35 cycles of denaturation at 95°C for 30 s, annealing at 49°C for 30 s and elongation at 72°C for 30 s, and a final elongation at 72°C for 5 min. Amplifications conditions for crystal protein (*Cry* 2) were: initial denaturation at 95°C for 5 min, followed by 35 cycles of denaturation at 95°C for 30 s, annealing at 55°C for 30 s and elongation at 72°C for 1 min, and a final elongation at 72°C for 5 min. After DNA amplifications, 1.5% (W/V) agarose gel solution containing Redsafe^TM^ Nucleic Acid Solution 20,000 × (Intron Biotechnology, Inc.) was prepared and gel electrophoresis was performed using Sub-Cell Model 192 from Bio-Rad located in Hemel Hempstead, United Kingdom. The bands were visualized using Smart View Pro UVCI-1100 Imager system from Major Science located in CA, United States. The accuracy (AC) was used to evaluate PCR method using primers *XRE* and *cry*2 in detecting *B. thuringiensis* from Bcg and non-B. Negative agreement (NA) means the same negative result for non*-B. thuringiensis* using PCR with *XRE* and *cry*2. Positive agreement (PA) means the same positive result for *B. thuringiensis* using PCR with *XRE* and *cry*2. The accuracy (AC) is measured using the following equation, AC = [(PA+NA)/N] ×100% (Ma et al., 2014).

Real-time PCR experiments were performed by the StepOne^TM^ real-time PCR System from Applied Biosystems located in CA, United States. A reaction volume of 20 μL consisting of 10 μL of Melt Doctor^TM^ High Resolution Melting (HRM) Master Mix, 2 μL of DNA, 0.5 μL of the F/R primer (10 pmoL/μL) (Table 2). Amplification conditions of the qPCR were: initial denaturation at 95°C for 10 min and 40 cycles at 95°C for 15 s and 60°C for 1 min. Then, in the subsequent melting curve analysis, the temperature was set to raise from 60 to 95°C at 0.3°C/cycle to test the specificity of the primer.

**Table 2:** Detection of *B. cereus* group and non-*Bacillus* sp. targeting biomarkers of *XRE* and *cry2* using PCR.

Specific markers	Parameter^a^	*B. cereus* (Bc)	*B. thuringiensis* (Bt)	Other *B. cereus* group srains (Bcg)	Non-*Bacillus* sp. (nonB)	Both Bc and Bt	*B. cereus* group (Bc, Bt, and Bcg)	Bc, Bt, Bcg, and non-B
Transcriptional regulator (*XRE*)	NA	58	0	3	9	58	61	70
	PA	0	47	0	0	47	47	47
	N	58	50	3	9	108	111	120
	AC (%)	100	94	100	100	97.2	97.3	97.5
Crystal protein (*cry*2)	NA	52	0	3	9	52	55	64
	PA	0	36	0	0	36	36	36
	N	58	50	3	9	108	111	120
	AC (%)	89.6	72	100	100	81.5	82	83.3


*^*a*^N means the total number of samples. AC (accuracy, 100%) = [(PA+NA)/N] × 100%.*

### Evaluation of Real-Time PCR Assay

The performance of *XRE* and *cry*2 was evaluated using 50 *B. thuringiensis* strains, while the exclusivity was tested using 70 non-target bacteria, including 58 *B. cereus* (Bc), 3 Bcg, and 9 non-B (Chelliah et al., 2017). A standard curve was made using 10-fold dilutions of the DNA extracted from *B. thuringiensis* ATCC 10792. DNA concentrations were adjusted from 10 ng/μL to 100 fg/μL, corresponding to 2.6 × 10^6^ to 2.6 × 10 CFU, and the amplifications were performed by real-time PCR with triplicates. For negative controls, distilled water was used. Negative results or no amplification curves were considered when the cycle threshold (Ct) values were more than 40.

### Detection of *B. thuringiensis* in Spiked Food Samples

Lettuce (*Lactucasativa*), Kimbab, and spinach (*Spinaciaoleracea*) were bought from a local market in Chuncheon, Korea. The samples were tested for the absence of target bacteria according to ISO 7932 (2004). An aliquot of 50 g of each kind of food was prepared in a sterile stomacher bag from Nasco Whirl-Pak located in WI, United States. Overnight *B. thuringiensis* cultures were diluted in 0.5% peptone water and used for preparing the artificially contaminated samples with inoculation levels from 10^3^ to 10^5^ CFU/g (Chon et al., 2012; Kim et al., 2013). An aliquot of 1 mL of the suspension of food samples was transferred to a new sterilized tube and used for DNA extraction.

## Results and Discussion

### Detection of *B. thuringiensis* Using *cry2* Gene

**FIGURE 1:**
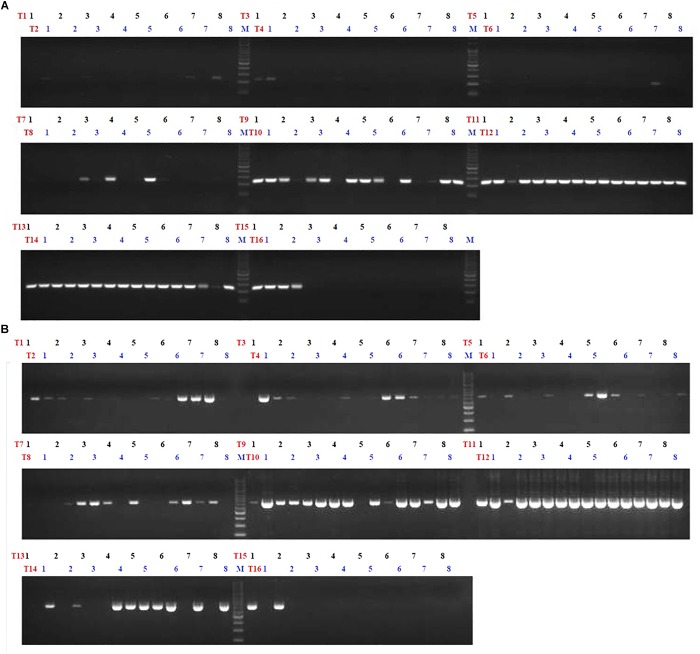
Conventional PCR results using 2 primers (*XRE* and *cry*2) targeting 2 genes (transcriptional regulator and crystal protein genes) specific for *B. thuringiensis* with 120 strains. **(A)** Is the PCR results of the 120 strains using *XRE* and **(B)** is the PCR results using *cry-*2. Lane M, 100 bp ladder; T1–T16 means the number of 8-strip tube and the details of each lane is shown in the Supplementary Table 1.

For the 120 strains, amplification products of approximately 700 bp were obtained using the primers *cry*2 (Table 1). As shown in Table 2 and Figure 1, when using PCR targeting *cry*2, 6 *B. cereus* strains and 36 *B. thuringiensis* strains (72%) were amplified. No other *B. cereus* group or non-B group was amplified. This showed an accuracy of 82% of *cry*2 for detecting *B. cereus groups*. For the 120 strains tested, 100 strains gave NAs or PAs, indicating an overall accuracy percentage of 83.3% (Supplementary Table 1). While Riojas et al. (2015) reported a multiplex PCR of non-ribosomal peptide synthase (NRPS) gene and *cry*1 to identify *B. cereus* and *B. thuringiensis* with a specificity of 24.5% in *B. cereus* and 15% in *B. thuringiensis*.

It has been reported that the *B. thuringiensis* strain may synthesize one or more crystal proteins since one or more crystal toxin genes have been found for *B. thuringiensis*. The main reason for the diversity of toxin genes is the transfer of plasmids in *B. thuringiensis* (Adang et al., 2014). It is a frequent process for the plasmid transfer by either conjugation or mobilization (Makart et al., 2017). Further, the exchanges of *cry* genes generate *B. cereus* with a new binding of *cry* that leads to the similarity of *B. cereus* and *B. thuringiensis* (Fiuza, 2015). In addition, with a different isolation source, the *cry* genes showed a difference in diversity and distributions. For instance, a novel *cry* gene can be found in each habitat that shows different insecticidal activity.

### Detection of *B. thuringiensis* Using *XRE* Gene

Potential coding sequences (CDS) were predicted to be transcriptional regulators. CDS contains a helix-turn-helix (HTH) motif in the *XRE*-like protein family and MerR family transcriptional regulators, previously the corresponding *XRE* gene sequences in pBMB28 of *B. thuringiensis* YBT-020 and pCT281 of *B. thuringiensis* (GenBank: CP001910). CDS50, which is related to the HTH-type transcriptional regulator, SinR, was identical to the corresponding element pG9842 of *B. cereus* G9842 (GenBank: CP001187). The HTH proteins, together with sigma factors, participate in a wide range of signaling pathways, for example, as repressors that inhibit sporulation (Murawska et al., 2014), biofilm formation (Colledge et al., 2011), and protease secretion (Pflughoeft et al., 2011). Apart from Cry1Ab21 and δ-endotoxin encoded in the toxigenic plasmid pIS56-63, which are responsible for conjugation and sporulation (Martínez-Núñez et al., 2010; Deng et al., 2014) in addition with 53 different encoded putative proteins.

The PCR results with *XRE* revealed that none of the 58 *B. cereus* or non-B gave an amplification (Table 2 and Figure 1). In total, out of 50 strains of *B. thuringiensis* 47 showed positive results (94%). This showed an accuracy of 97.3% of *XRE* for detecting *B. thuringiensis* among the tested *B. cereus* groups. For the 120 strains tested, 117 strains gave NAs or PAs, indicating an overall accuracy percentage of 97.5% (Supplementary Table 1).

### Standard Curve and Amplification Efficiency

**FIGURE 2:**
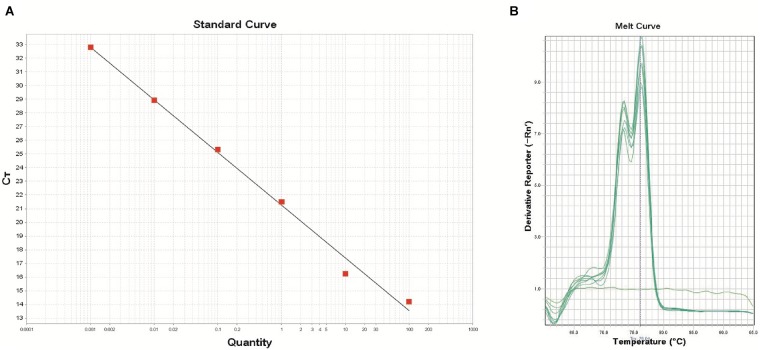
Standard curve **(A)** using the primer *XRE* (*R*^2^: 0.993, Eff%: 81.8) and melt curve peaks **(B)** for the detection of the transcriptional regulator and crystal protein genes in the *B. thuringiensis* strain. The DNA concentrations of *B. thuringiensis* ATCC 10792 were as follows: 1.0 × 10^2^, 1.0 × 10^1^, 1.0 × 10^0^, 1.0 × 10^-1^, 1.0 × 10^-2^, and 1.0 × 10^-3^ng (from left to right). DNA amounts correspond to 2.6 × 10^6^, 2.6 × 10^5^, 2.6 × 10^4^, 2.6 × 10^3^, 2.6 × 10^2^, and 2.6 × 10^1^ CFU, respectively.

The real-time PCR assay was performed using the DNA extracts from the pure cultures of *B. thuringiensis* type strain ATCC 10792 targeting the *XRE* gene. The developed real-time PCR assay was efficient for detecting concentrations from 2.6 × 10 to 2.6 × 10^6^ CFU/reaction, which covered 6 orders of magnitude. The equation of log copy number versus the Ct value for *B. thuringiensis* was y = -3.853x+21.244 with an R-squared value of 0.993, indicating the high linearity (Figure 2).

### Spiked Food Samples Detection

**FIGURE 3:**
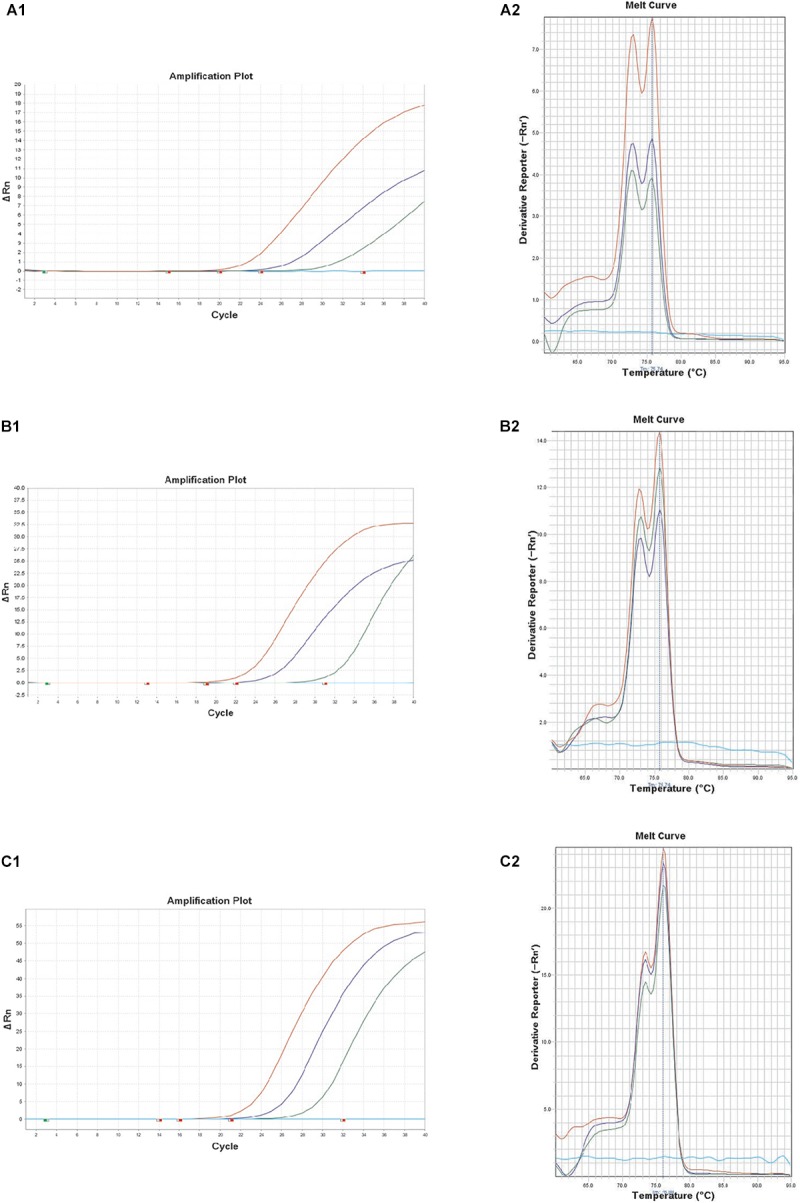
Melt curve peaks and amplification curves of *XRE* real-time PCR with *B. thuringiensis* ATCC 10792 in artificially contaminated food samples of lettuce **(A1,A2)**; spinach **(B1,B2)**; and kimbap **(C1,C2).** Colony forming units of the amplification curves correspond to 8.4 × 10^2^, 8.4 × 10^1^, 8.4 × 10^0^ CFU (lettuce), 9.0 × 10^2^, 9.0 × 10^1^, 9.0 × 10^0^ CFU (spinach), and 2.4 × 10^2^, 2.4 × 10^1^, 2.4 × 10^0^ CFU (kimbap), respectively.

Recently, *B. thuringiensis* have been reported to be detected in foods such as milk, fresh fruits and grains that have been cultivated and harvested from soil outside the country. The consumption of raw and minimally processed vegetables is considered natural and healthy but concerns regarding residual chemical pesticides in fresh vegetables have been raised (Chiu et al., 2016). Thus, consumers have turned their interest to organic vegetables (chemical free vegetables), and it is predicted that bio-pesticides, including *B. thuringiensis*, may account for 20% of the world’s pesticide market by 2020 as a substitute for chemical pesticides (Watts and Williamson, 2015). In this study, the performance of the real-time PCR targeting *XRE* was evaluated with 3 kinds of artificially contaminated food samples (lettuce, kimbap and spinach). Fresh overnight cultures of *B. thuringiensis* were inoculated into each food sample. The real-time PCR could successfully detect *B. thuringiensis* at a concentration of 4.8 × 10^3^, 3.4 × 10^3^, and 1.5 × 10^3^ CFU/g in lettuce, kimbap and spinach, respectively (Figure 3). However, for kimbap samples, the Ct values were higher, since usually it contains more complex materials such as sausage, egg or carrot compared with the vegetables.

## Conclusion

Since *B. cereus* group are highly similar in biochemical as well as genetic profiles, a new biomarker was developed for identifying and distinguishing *B. thuringiensis* from the closely related group. The performance of *XRE* was compared with *cry*2 gene and artificially contaminated samples were also tested. Compared with *cry*2, *XRE* gene was observed to be efficiently accurate in the identification of *B. thuringiensis.* Further, the developed real-time PCR using *XRE* successfully identified *B. thuringiensis* and it could be used to quantify cell numbers with the generated standard curve.

## Author Contributions

The experiments were conceived and designed by SW, RC, YG-J, and DH-O. SW, BJ-P, FF, MC, and DS-P performed the experiments. BJ-P and SH-K discussed the experiments and results. SW and RC wrote the manuscript.

## Conflict of Interest Statement

The authors declare that the research was conducted in the absence of any commercial or financial relationships that could be construed as a potential conflict of interest.
