# Supplementary material for: Differentiation of Bacillus thuringiensis From Bacillus cereus Group Using a Unique Marker Based on Real-Time PCR
Source: Front Microbiol. 2019 Apr 30;10:883. doi: 10.3389/fmicb.2019.00883 (PMC6503103; doi:10.3389/fmicb.2019.00883)
Supplement: Supplementary file 1 [file Table_1.docx]

**Differentiation of *Bacillus thuringiensis* from *Bacillus cereus* group using a unique marker based on real-time PCR**

Shuai Wei^1†^, Ramachandran Chelliah^2†^, Byung-Jae Park^2^, Se-Hun Kim^c^, Fereidoun Forghani^3^, Min Seok Cho^4^, Dong-Suk Park^4^, Yong-Guo Jin^5*^, Deog-Hwan Oh^2*^

**S Table 1 PCR results of the 120 strains**

| 8-strip -tube # | Strain names | PCR result^a^ | |
| --- | --- | --- | --- |
|  |  | *XRE* | *cry2* |
| 01-01 | B. cereus ATCC 14579 | - | - |
| 01-02 | B. cereus ATCC 27348 | - | - |
| 01-03 | B. cereus IAM 1729; KCTC1094; KACC12682 | - | - |
| 01-04 | B. cereus NRRL B-569; KCTC 1526 | - | - |
| 01-05 | B. cereus ATCC 10876; KCTC 1092 | - | - |
| 01-06 | B. cereus ATCC 12480 | - | - |
| 01-07 | B. cereus F4810/72 | - | + |
| 01-08 | B. cereus ATCC 21768; KCTC 1014 | - | - |
| 02-01 | B. cereus KACC 13064 | - | - |
| 02-02 | B. cereus ATCC 14893; NCCP 10623 | - | - |
| 02-03 | B. cereus ATCC 21928; NCCP 10715 | - | - |
| 02-04 | B. cereus ATCC 14579; NCCP 14579 | - | - |
| 02-05 | B. cereus ATCC 11778; KCCM 11204 | - | - |
| 02-06 | B. cereus ATCC 13061 | - | + |
| 02-07 | B. cereus ATCC 21366; KCTC 1013 | - | + |
| 02-08 | B. cereus ATCC 25621 | - | - |
| 03-01 | B. cereus F4433/73 (diarrheal) | - | - |
| 03-02 | B. cereus KFDA wild type 583 | - | - |
| 03-03 | B. cereus KFDA wild type 584 | - | - |
| 03-04 | B. cereus KFDA wild type 585 | - | - |
| 03-05 | B. cereus KFDA 229 | - | - |
| 03-06 | B. cereus KFDA 250 emetic | - | - |
| 03-07 | B. cereus KUGH 12 emetic | - | - |
| 03-08 | B. cereus KUGH 27 emetic | - | - |
| 04-01 | B. cereus KUGH 85 emetic | - | + |
| 04-02 | B. cereus 617-1 | - | - |
| 04-03 | B. cereus 728-1 | - | - |
| 04-04 | B. cereus 86-2 | - | - |
| 04-05 | B. cereus B21A wild type isolate | - | - |
| 04-06 | B. cereus B9A wild type isolate | - | + |
| 04-07 | B. cereus 62-2 | - | - |
| 04-08 | B. cereus 72-1 | - | - |
| 05-01 | B. cereus B.C.-0001 | - | - |
| 05-02 | B. cereus B.C.-0002 | - | - |
| 05-03 | B. cereus B.C.-0005 | - | - |
| 05-04 | B. cereus ICHE-1 | - | - |
| 05-05 | B. cereus ICHE-2 | - | - |
| 05-06 | B. cereus ICHE-3 | - | - |
| 05-07 | B. cereus ICHE-4 | - | - |
| 05-08 | B. cereus JNHE 06 | - | - |
| 06-01 | B. cereus JNHE 13 | - | - |
| 06-02 | B. cereus JNHE 15 | - | - |
| 06-03 | B. cereus JNHE 22 emetic | - | - |
| 06-04 | B. cereus KFDA 202 | - | - |
| 06-05 | B. cereus KFDA 203 | - | + |
| 06-06 | B. cereus KFDA 204 | - | - |
| 06-07 | B. cereus KFDA 205 | - | - |
| 06-08 | B. cereus KNIH ulsan 1 emetic | - | - |
| 07-01 | B. cereus KNIH ulsan 2 emetic | - | - |
| 07-02 | B. cereus KNIH ulsan 3 emetic | - | - |
| 07-03 | B. cereus KNIH ulsan 4 emetic | - | - |
| 07-04 | B. cereus KUGH-B.C emetic 1 | - | - |
| 07-05 | B. cereus KUGH-B.C emetic 5 | - | - |
| 07-06 | B. cereus KUGH-B.C emetic 4 | - | - |
| 07-07 | B. cereus KUGH-B.C emetic 7 | - | - |
| 07-08 | B. cereus 4153 | - | - |
| 08-01 | B. cereus 4154 | - | - |
| 08-02 | B. cereus 4158 | - | - |
| 08-03 | B. cereus 4165 | - | - |
| 08-04 | B. cereus ATCC 14579 as PC^b^ | - | - |
| 08-05 | B. thuringiensis ATCC 10792 as PC | + | - |
| 08-06 | E. coli ATCC 43895 as NC | - | - |
| 08-07 | S. aureus ATCC 12600 as NC | - | - |
| 08-08 | Blank (no gDNA) | - | - |
| 09-01 | B. thuringiensis ATCC 10792; KACC12061 | + | - |
| 09-02 | B. thuringiensis HD-73; KCTC 1508 | + | + |
| 09-03 | B. thuringiensis ATCC 13367; KACC 14395 | + | + |
| 09-04 | B. thuringiensis serovar tolworthi NRRL HD-13 | + | + |
| 09-05 | B. thuringiensis serovar indiana NRRL HD-516 | + | - |
| 09-06 | B. thuringiensis serovar morisoni NRRL HD-12 | - | - |
| 09-07 | B. thuringiensis KCTC 1510 | + | + |
| 09-08 | B. thuringiensis subsp. kurstaki CAB141 | + | + |
| 10-01 | B. thuringiensis subsp. kurstaki HD-1 | + | + |
| 10-02 | B. thuringiensis subsp. israelensis H14 | + | + |
| 10-03 | B. thuringiensis subsp. aizawai CAB109 | + | + |
| 10-04 | B. thuringiensis subsp. aizawai H7 | + | + |
| 10-05 | B. thuringiensissubsp.aizawaiNT0423 | + | + |
| 10-06 | B. thuringiensissubsp.aizawaiGB413 | + | + |
| 10-07 | B. thuringiensis nontoxic (KA4-1) | - | - |
| 10-08 | B. thuringiensis nontoxic (CAB506) | + | + |
| 11-01 | B. thuringiensis nontoxic (KA2-2) | + | + |
| 11-02 | B. thuringiensis CAB101 | - | - |
| 11-03 | B. thuringiensis CAB104 | + | + |
| 11-04 | B. thuringiensis CAB105 | + | + |
| 11-05 | B. thuringiensis CAB106 | + | + |
| 11-06 | B. thuringiensis CAB107 | + | + |
| 11-07 | B. thuringiensis CAB108 | + | + |
| 11-08 | B. thuringiensis CAB110 | + | + |
| 12-01 | B. thuringiensis CAB120 | + | + |
| 12-02 | B. thuringiensis CAB125 | + | + |
| 12-03 | B. thuringiensis CAB-128 | + | + |
| 12-04 | B. thuringiensis CAB130 | + | + |
| 12-05 | B. thuringiensis CAB135 | + | + |
| 12-06 | B. thuringiensis CAB136 | + | + |
| 12-07 | B. thuringiensis CAB137 | + | + |
| 12-08 | B. thuringiensis CAB138 | + | + |
| 13-01 | B. thuringiensis CAB139 | + | - |
| 13-02 | B. thuringiensis CAB140 | + | - |
| 13-03 | B. thuringiensis CAB143 | + | - |
| 13-04 | B. thuringiensis CAB155 | + | - |
| 13-05 | B. thuringiensis CAB156 | + | + |
| 13-06 | B. thuringiensis CAB159 | + | + |
| 13-07 | B. thuringiensis CAB162 | + | - |
| 13-08 | B. thuringiensis CAB191 | + | - |
| 14-01 | B. thuringiensis CAB255 | + | - |
| 14-02 | B. thuringiensis CAB280 | + | - |
| 14-03 | B. thuringiensis CAB315 | + | - |
| 14-04 | B. thuringiensis CAB318 | + | + |
| 14-05 | B. thuringiensis CAB420 | + | + |
| 14-06 | B. thuringiensis CAB430 | + | + |
| 14-07 | B. thuringiensis CAB431 | + | + |
| 14-08 | B. thuringiensis CAB435 | + | + |
| 15-01 | B. thuringiensis CAB450 | + | + |
| 15-02 | B. thuringiensis CAB461 | + | + |
| 15-03 | Blank (no gDNA) | - | - |
| 15-04 | B. mycoides ATCC 6462 | - | - |
| 15-05 | B. pseudomycoides NRRLB-617 | - | - |
| 15-06 | B. weihenstephanensis KACC12001 | - | - |
| 15-07 | B. amyloliquefaciens CJ3-27 | - | - |
| 15-08 | B. subtilis ATCC 6051 | - | - |
| 16-01 | E. coli ATCC 43895 | - | - |
| 16-02 | Enterococcus feculis | - | - |
| 16-03 | L. monocytogenes ATCC 15313 | - | - |
| 16-04 | Micrococcus luteus ATCC 10240 | - | - |
| 16-05 | Salmonella enterica ATCC 13076 | - | - |
| 16-06 | S. aureus ATCC 12600 | - | - |
| 16-07 | S. pyogenes ATCC 12348 | - | - |
| 16-08 | Blank (no gDNA) | - | - |

^a^ The symbol “+” means a clear band was observed in the gel under UV light indicating a positive result and “-” means no band was observed in the gel under UV light indicating a negative result.

^b^ PC means positive control. NC means negative control.
